# Potential secondary metabolite from Indonesian Actinobacteria (InaCC A758) against *Mycobacterium tuberculosis*

**DOI:** 10.22038/ijbms.2021.56468.12601

**Published:** 2021-08

**Authors:** Maya Dian Rakhmawatie, Tri Wibawa, Puspita Lisdiyanti, Woro Rukmi Pratiwi, Ema Damayanti, Mustofa

**Affiliations:** 1 Doctoral Program in Faculty of Medicine, Public Health and Nursing, Universitas Gadjah Mada, Yogyakarta 55281, Indonesia; 2 Department of Biomedical Sciences, Faculty of Medicine, Universitas Muhammadiyah Semarang, Semarang 50273, Indonesia; 3 Department of Microbiology, Faculty of Medicine, Public Health and Nursing, Universitas Gadjah Mada, Yogyakarta 55281, Indonesia; 4 Research Center for Biotechnology, Indonesian Institute of Sciences, Kabupaten Bogor, West Java 16911, Indonesia; 5 Department of Pharmacology and Therapy, Faculty of Medicine, Public Health and Nursing, Universitas Gadjah Mada, Yogyakarta 55281, Indonesia; 6 Research Division of Natural Product Technology, Indonesian Institute of Sciences, Yogyakarta 55861, Indonesia

**Keywords:** Actinobacteria, Dactinomycin, Dimethenamid, Mass spectrometry, Mycobacterium tuberculosis, Peptide synthases RNA, Ribosomal, 16S Streptomyces

## Abstract

**Objective(s):**

This study explored Indonesian Actinobacteria which were isolated from *Curcuma zedoaria* endophytic microbes and mangrove ecosystem for new antimycobacterial compounds.

**Materials and Methods:**

Antimycobacterial activity test was carried out against *Mycobacterium tuberculosis *H37Rv. Chemical profiling of secondary metabolite using Gas Chromatography-Mass Spectroscopy (GC-MS) and High Resolution-Mass Spectroscopy (HR-MS) was done to the ethyl acetate extract of active strain InaCC A758. Molecular taxonomy analysis based on 16S rRNA gene and biosynthetic gene clusters analysis of polyketide synthase (PKS) and non-ribosomal peptide synthetase (NRPS) from InaCC A758 have been carried out. Bioassay guided isolation of ethyl acetate extract was done, then structural elucidation of active compound was performed using UV-Vis, FT-IR, and NMR spectroscopy methods.

**Results:**

The chemical profiling using HR-MS revealed that InaCC A758 has the potential to produce new antimycobacterial compounds. The 16S rRNA gene sequencing showed that InaCC A758 has the closest homology to *Streptomyces parvus *strain NBRC 14599 (99.64%). In addition, InaCC A758 has NRPS gene and related to *S. parvulus* (92% of similarity), and also PKS gene related to PKS-type borrelidin of S. rochei and *S. parvulus *(74% of similarity). Two compounds with potential antimycobacterial were predicted as 1) Compound 1, similar to dimethenamid (C_12_H_18_ClNO_2_S; MW 275.0723), with MIC value of 100 µg/ml, and 2) Compound 2, actinomycin D (C_6_2H_86_N_12_O_16_; MW 1254.6285), with MIC value of 0.78 µg/ml.

**Conclusion:**

Actinomycin D has been reported to have antimycobacterial activity, however the compound has been predicted to resemble dimethenamid had not been reported to have similar activity.

## Introduction

Tuberculosis (TB) is a contagious infectious disease caused by *Mycobacterium tuberculosis*. This disease primarily affects the lungs and can be transmitted through bacteria-containing air (1). World Health Organization (WHO) stated that in 2018 there were 417.000–556.000 new Rifampicin Resistance (RR-TB) cases with 78% of them developed into Multi-Drug Resistance (MDR-TB). Even today, 128 countries around the world reported at least one case of Extensively Drug-Resistant (XDR-TB). The existence of MDR/RR-TB and XDR-TB cases is one of the reasons for the need new antimycobacterial drugs, although the previous drug combination regimen can be still used (2, 3). Another reason for the urgent need of new antimycobacterial drugs is the fact that since the 1960s there were only a little progress in the availability of new drugs for TB infection, such as streptomycin in 1944 and bedaquiline that was approved by the FDA in 2012 (4).

To fulfill the need for new antimycobacterial, some efforts have been done, such as finding compounds from natural products or synthetic compounds. Today, natural compounds contribute 45% of total drug sales. In addition, with the development of bioinformatics and metagenomics technology, the discovery of natural compounds-derived drugs re-enter the golden age, as happened during 1950-1960s (5, 6). One of the strategies to find new natural compounds is by exploring secondary metabolites produced by Actinobacteria. So far, several potential actinobacteria have been found to produce new antimycobacterial compounds. Some of them are the discovery of cyclic peptide compounds which is also a potential anti-MDR-TB and XDR-TB *in vitro* (7), and also the discovery of antimycobacterial compounds from marine *Streptomyces *sp*. *MS100061, including spirotetronate, lobophorin G, lobophorin A, and lobophorin B (8).

Indonesia is a biodiverse country. The biodiversity includes abundant microorganisms such as Actinobacteria from soil or water samples (9). However, the exploration of actinobacteria isolated from Indonesia as a producer of antimycobacterial compounds has not been widely carried out. This study explored actinobacteria isolated from *Curcuma zedoaria* endophytic microbes as well as from the mangrove system in Pramuka Island, Kepulauan Seribu, Indonesia. The discovery of antibacterial from endophytic microbes (10) or actinobacteria isolated from the mangrove system has been reported previously (11). Meanwhile, the diversity of actinobacteria isolated from *Curcuma zedoaria* or the mangrove system might be potential to discover new antibacterial compounds (12, 13).

During the study on actinobacteria screening, one of the isolates, InaCC A758, has the potential to produce active compounds inhibiting the growth of *M tuberculosis* strain H37Rv. Taxonomically based on the 16S rRNA gene, as well as the gene clusters producing secondary metabolites polyketide synthase (PKS) and non-ribosomal peptide synthetase (NRPS) carried out on the InaCC A758. Metabolite profiling results of InaCC A758 ethyl acetate extract using Gas Chromatography-Mass Spectroscopy (GC-MS) (14) and High Resolution-Mass Spectroscopy (HR-MS) (15) were presented, and can be used to see the diversity of secondary metabolites produced and their novelty. The bioassay-guided isolation process followed by a structural elucidation using UV-Vis, Fourier-transform infrared (FT-IR), and Nuclear Magnetic Resonance (NMR) spectroscopy was carried out, to ensure active compounds that have antimycobacterial activity from the InaCC A758.

## Materials and Methods

***Actinobacteria and M. tuberculosis strains***

The sixteen Actinobacteria used in this research were obtained from the Indonesian Culture Collection (InaCC), Indonesian Institute of Sciences, West Java, Indonesia. While, *M. tuberculosis* strain H37Rv was obtained from Microbiology Laboratory, Faculty of Medicine, Public Health and Nursing, Universitas Gadjah Mada, Yogyakarta, Indonesia. This strain is used in this antimycobacterial screening because of its moderate growth. *Mycobacterium tuberculosis* was grown on Lowenstein Jensen (LJ) agar and used for the screening after incubated at 37 °C for 3-4 weeks (16).

***Secondary metabolite production and extraction***

Actinobacteria were pre-cultured in Starch Yeast Peptone (SYP) broth media (contain 10.0 g starch (Merck, Germany), 4.0 g yeast extract (BD Difco, USA), and 2.0 g bacto peptone (BD Difco, USA) per 1-liter water) for 48 hr. Ten percent volume of pre-culture was transferred to a baffled flask containing new SYP broth media as production medium, and the total amount of culture media was 20% of the maximum volume of the baffled flask used (17). The incubation temperature was 30 °C, shaker orbital agitation speed was 135 rpm, and cultured was done in 72 hr. The supernatant of culture suspension was separated from cell biomass using centrifugation at 6000 rpm, 15 min, 22 ºC. The supernatant was then extracted by the addition of 1:1 v/v ethyl acetate. The crude ethyl acetate extract was collected then dried using a rotary evaporator (Buchi, Switzerland) at 40 °C (18).

***Antimycobacterial screening of actinobacteria extracts***

The screening procedure for the antimycobacterial activity of Actinobacteria extracts was carried out using the REMA and CVDA methods and had been described in previous studies (19). The maximum concentration was diluted by the two-fold dilutions method from 1.0 to 0.0625 µg/ml for isoniazid and rifampicin, as well as from 100.0 to 6.25 µg/ml for Actinobacteria extract. 

***Amplification and sequencing of 16S rRNA, NRPS, and PKS gene from actinobacteria InaCC A758***

InaCC A758 were first cultured using Tryptic Soy Broth (BD Difco, USA) media, 48 hr at 30 °C of temperature, then DNA extraction was done using Purelink^TM^ Genomic DNA Minikit (Invitrogen K1821-04). For 16S rRNA gene amplification, primer 9-F and 1541-R were used (20)iterative, hybrid, and enediyne polyketide synthases (PKSs and the PCR mixture was carried out using the standard method of the kit (Gotaq® Green Master Mix, Promega M7122). Amplification was carried out with 5 min pre-denaturation at 96 °C; continued with 30 cycles of 30 sec denaturation at 96 °C, 30 sec annealing at 55 °C, 1-min elongation at 72 °C, and followed by final extension for 7 min with the temperature at 72 °C. 

For amplification of type I PKS gene, a 50 µl PCR mixture was prepared which was consisted of 1 µl DNA, 2 µl each primer K1-F and M6-R (21) with a concentration of 10 µM, 20 µl ddH_2_O, and 25 µl PCR master mix (Gotaq® Green Master Mix, Promega M7122). Amplification was carried out with 5 min pre-denaturation at 95 °C; continued with 30 cycles of 30 sec denaturation at 95 °C; 40 sec annealing at 55 °C; 2 min elongation at 72 °C, and followed by final extension for 10 min at 72 °C. For amplification of NRPS gene, the mixture consisted of 1 µl DNA, 2.5 µl each primer A3-F and A7-R (21) with a concentration of 10 µM, 20 µl ddH_2_O, and 24 µl PCR master mix (Gotaq® Green Master Mix, Promega M7122). Amplification was carried out using the same method as the PKS gene, but required a longer annealing time (2 min).

Sequencing of amplification product from 16S rRNA, PKS, and NRPS genes was performed using each original primer and was done by a Genetika Science Sequence Service Company, Tangerang, Indonesia. For sequence data assembling, the BioEdit version 7 program was used. A homology sequence data match was performed using BLAST nucleotide for 16S rRNA gene, and BLASTx for PKS and NRPS gene, available online at https://blast.ncbi.nlm.nih.gov/Blast.cgi. The phylogenetic tree was constructed by MEGA 7.0 software using the Neighbor-Joining Method (22).

***Chemical profiling using GC-MS and HR-MS***

The chemical profiling was first performed using GC-MS. The ethyl acetate extract of InaCC A758 was dissolved using methanol to a concentration of 1 mg/ml and injected as much as 10 μl. The column used was TG-5MS 30 m x 0.25 mm x 0.25 μm (Thermo Fischer Sci TF26097-1420), with injector temperature 230 °C. Helium was used as a gas with a flow rate of 1 ml/min. The oven temperature was set at 60 °C for 2 min, then was gradually risen 10 °C/min and was held at 280 °C for 8 min. The total separation time was 31.96 min, and the m/z scan was in the range of 30.00 - 500.00. The chromatogram peak data analysis was performed by comparing the NIST 14 GC-MS Library database (23).

Further chemical profiling was performed using HR-MS, UHPLC (Thermo Scientific™ Ultimate™ 3000 RSLCnano) was used and coupled with mass detector (Thermo Scientific™ Q Exactive™ High-Resolution Mass Spectrometer). The column used in compounds separation using HR-MS was analytical column C_18_ 1.9 µm; 1 x 50 mm (Hypersil GOLD^TM^ aQ). Ethyl acetate extract of InaCC A758 was prepared at a concentration of 4 mg/ml, and the number of samples injected into the column is 5 µl. The mobile phases used were A (0.1% formic acid in H_2_O) and B (0.1% formic acid in acetonitrile), and the flow rate was 10 µl/min with a gradient flow of 5 - 95% B for 22 min, then the flow rate 95% B was held for 3 min, and then returned at a 5% B after 25.1 min to 30 min. Ionization used on MS scans was heated ESI (+) with full MS at 70000 FWHM, data-dependent MS2 at 17500 FWHM. Secondary metabolite compounds in the ethyl acetate extract of InaCC A758 were identified using Thermo Scientific^®^ Compound Discoverer Software.

***Bioassay-guided isolation***

The fractionation of ethyl acetate extract of InaCC A758 was carried out using a semi-preparative HPLC (binary HPLC pump, Waters 1525) coupled with Photo Diode Array (PDA) detector (Waters 2998), and C_18_ column 5 µm; 7.8 x 150 mm (Xterra^®^ Prep MS). The mobile phase system used was a gradient elution from 10 to 100% acetonitrile in water within 20 min. The mobile phase flow rate used was 1 ml/min, and λ 200 - 600 nm used for peak chromatogram detection. To test the activity of the fraction, the MIC determination was done using the same method as the antimycobacterial screening test. After an active fraction was obtained, the purification process was then performed using a semi-preparative HPLC with C_18_ column, with varieties of mobile phase systems from the gradient elution system to the isocratic elution system.

***Structural elucidation of active antimycobacterial compounds***

Simple UV-VIS and infrared spectroscopic methods (Nicolet^TM^ iS^TM^ 10) were used to determine the structure of the compounds. Purified fractions were mashed with 100 mg potassium bromide using agate mortar stamper, then were put into a pellet disc maker mold and were pressed using a hydraulic pump. Finished and clear discs were then inserted into the FTIR device to be scanned with a range of 4000 - 400 cm^-1^. 

Proton measurements were conducted by using a 500 MHz frequency of ^1^H-NMR (Jeol JNM-ECZ500R). Purified fractions were respectively dissolved with deutenetration CD_3_OD solvent. A 400 MHz frequency of ^13^C-NMR with 7000 scans was employed for carbon measurement. Additional NMR analyzes such as DEPT 135 were also conducted.

## Results

***Screening of antimycobacterial activity and*** ***detection of*** ***NRPS*** ***and*** ***PKS genes***

The sixteen Actinobacteria were screened for antimycobacterial activities. The results showed that one Actinobacteria produced compounds that were able to inhibit the growth of *M. tuberculosis* strain H37Rv, namely InaCC A758 (MIC 25.0 µg/ml). The control drugs used in this study were isoniazid and rifampicin, both could inhibit the growth of *M. tuberculosis* with MIC value of 0.0625 and 0.125 µg/ml, respectively. Actinobacteria InaCC A758 was isolated from rhizosphere soil (mud) of mangrove area in Pramuka Island, Indonesia. The isolate was detected possessing NRPS and PKS cluster genes (Table 1).

***Sequencing and phylogenetic analysis of 16S rRNA, PKS, and NRPS genes of InaCC A758***

**Figure 1:**
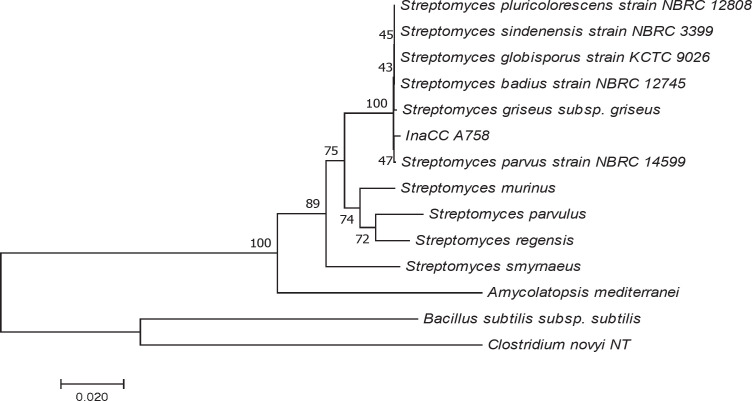
InaCC A758 phylogenetic tree based on the sequence of 16S rRNA gene. The phylogenetic tree was constructed using the Neighbor-Joining method (24). The percentage of replications associated with the taxa cluster (1000 bootstrap test replications) is shown next to the branch line (25). The evolutionary distance was calculated using the Tamura-Nei method (26), and the degree of variation among isolates was modeled using gamma distribution. Alignment gaps, missing data, and ambiguous bases are allowed a maximum of 5% (22)

Based on 16S rRNA gene sequencing and phylogenetic tree analysis, InaCC A758 was closely related to *Streptomyces parvus* NBRC 14599 (NR_112437) (99.64%) (Figure 1). To the best our knowledge, there was no discovery of antimycobacterial compounds from *S. parvus* strain NBRC 14599 that had been reported yet.

**Figure 2:**
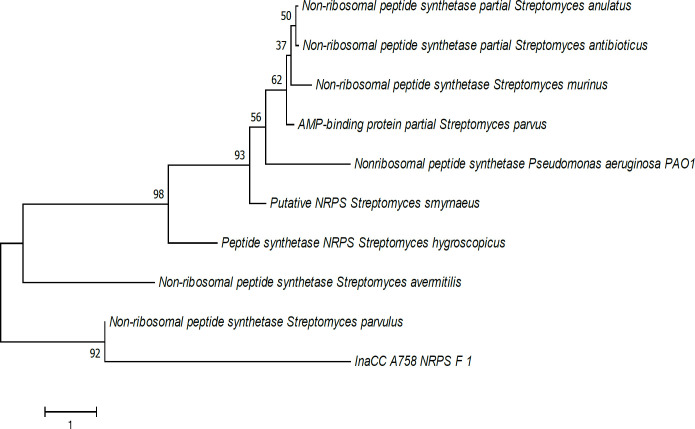
InaCC A758 phylogenetic tree based on the sequence of NRPS gene. Phylogenetic tree is constructed using Neighbor-Joining method (24). The percentage of replications associated with the taxa cluster (1000 bootstrap test replications) is shown next to the branch line (25). The evolutionary distance is calculated using the Poisson method, and the degree of variation among isolates was modeled using gamma distribution. Alignment gaps, missing data, and ambiguous bases are allowed a maximum of 5% (22)

**Figure 3:**
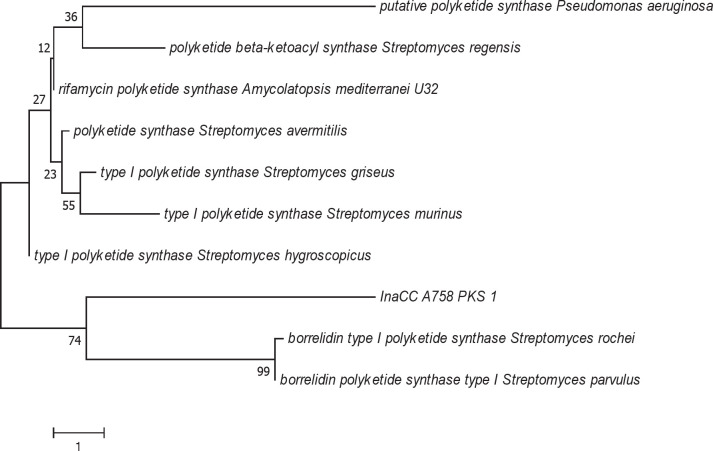
InaCC A758 phylogenetic tree based on the sequence of PKS gene. Phylogenetic tree is constructed using Neighbor-Joining method (24). The percentage of replications associated with the taxa cluster (1000 bootstrap test replications) is shown next to the branch line (25). The evolutionary distance is calculated using the Poisson method, and the degree of variation among isolates was modeled using gamma distribution. Alignment gaps, missing data, and ambiguous bases are allowed a maximum of 5% (22)

The NRPS gene of InaCC A758 showed a 92% correlation to the NRPS gene from *Streptomyces parvulus* (Figure 2). The PKS gene phylogenetic tree analysis of InaCC A758 indicated a 74% correlation to the type I PKS borrelidin gene from *Streptomyces rochei* and *S. parvulus* (Figure 3).

***Chemical profiling using GC-MS and HR-MS***

The metabolite profiling analysis of ethyl acetate extract of InaCC A758 was performed using GC-MS. Based on the chromatogram profile, several kinds of Fatty Acid Methyl Ester (FAME) compounds were identified, although it was not the main metabolite. The alkene compound octadecane, 6-methyl being the main metabolite of ethyl acetate extract of InaCC A758 (Table 2). 

Meanwhile, the secondary metabolite profiling using HR-MS revealed that ethyl acetate extract of InaCC A758 produces 13 peaks with a minimum area of 0.4%. Peaks similarity compared to the database were between 57.7 – 94.1 % (Table 3).

***Fractionation of active compounds from InaCC A758 ethyl acetate extract***

**Figure 4:**
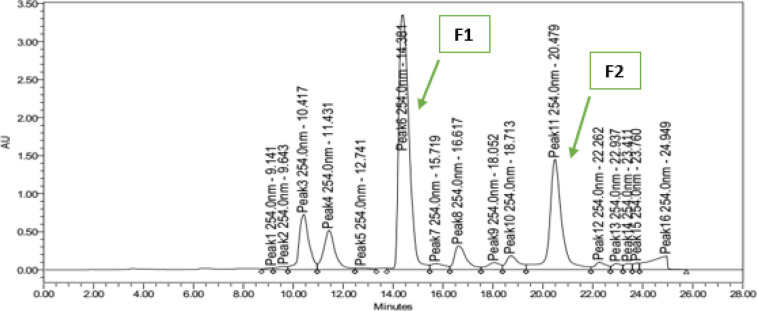
HPLC analysis of the ethyl acetate extract of strain InaCC A758. The extracts were analyzed using HPLC with semi-preparative column (7.2 µM, 4.6 x 150 mm), acetonitrile in water as mobile phase with a gradient elution concentration from 10 to 100% in 20 minutes, 1.0 ml/min as the flow rate, and 254 nm as the UV detection wavelength

A total of 16 fractions based on chromatogram peaks during the separation process were collected and dried. Of the 16 fractions, two fractions inhibited the growth of *M. tuberculosis*, namely the F1 fraction with a retention time (RT) of 14.381 min (MIC 100 µg/ml) and the F2 fraction with a retention time of 20.479 min (MIC 6.25 µg/ml) (Figure 4).

The active fractions F1 and F2 were then purified using preparative HPLC. The F1 fraction produces 2 chromatogram peaks, using an isocratic mobile phase of water:acetonitrile (40:60). Active peak (compound 1) appears in RT 6.381 min occupies an area of ​​95% (MIC 100 µg/ml). The F2 fraction produced 6 chromatogram peaks, using a linear gradient system of 10-100% acetonitrile in water for 20 min. Active peak (compound 2) appears in RT 21.082 min occupies an area of ​​87% (MIC 0.78 µg / ml).

***Structural elucidation of active compounds***

Compound 1 has a hint of purplish color when dissolved with methanol and white crystal powders when was dried; UV (CH_3_OH) l_max_ 340 nm. HR-MS data for possible compound 1, with an area of ​​50.72% (RT 15.95 min), was dimethenamid (C_12_H_18_ClNO_2_S, MW 275.0723), with a 57.7% similarity index compared to the database.

Compound 1 was predicted to have the following functional groups: methyl or methylene CH bonds (1464 cm-1), CC skeletal vibrations (1253 - 766 cm-1), aromatic rings (1464 cm-1), fluoro aliphatic (1129 - 1055 cm- 1), aliphatic chloro (793 - 766 cm-1), bromo aliphatic (691 cm-1), normal OH polymeric (3406 - 3200 cm-1), tertiary aromatic amines (1355 cm-1), amides (1658 cm- 1). There were 24 carbon peaks and ester groups detected with the ^13^C-NMR (400 MHz; CD_3_OD) analysis (). Whereas the ^1^H-NMR (500 MHz; CD_3_OD) detected an aromatic phenol or methyl aromatic group (Table 5).

Compound 2 was isolated as a red powder and turn into orange-yellow when dissolved with methanol; UV (CH_3_OH) l_max_ 240.6 and 440.1 nm. According to the HR-MS data, the prediction of compound 2 was actinomycin D (C_62_H_86_N_12_O_16, _MW 1254.6285) with an 82.5% similarity index compared to the database. 

Based on FT-IR data, compound 2 was predicted to have the following functional groups: C = O ester (1653 cm-1), and O–H acid or –NH amide group (3400 - 3500 cm-1). Based on the reading of ^1^H-NMR (500 MHz; CD_3_OD) data for compound 2 (Table 6), it can be concluded that there were several types of hydrogen. For example, δ 0.78–0.98 (d, methyl R-CH3), 1.28 (d, methylene R-CH2-), 1.14–4.86 (d, R-NH2), 7.45–7.54 (d, aromatic group), and 8.16 (d, RCONHR).

From the ^13^C-NMR (400 MHz; CD_3_OD) data for compound 2, seen several types of carbon; δ 15.17–39.51 (alkane groups; R-CH_3_, R-CH_2_, CH, C-), 49.15–58.64 (amino groups, R-NH_2_), 72.12–76.41 (C-O group), 126.55–149.11 (aromatic groups), and 168.31–180.74 (carbonyl group, either ester, amide, or carboxylic acid) (Table 7). Additional DEPT-135 analysis showed that there were several alkane groups (R-CH_2_). The peak which indicated the possibility of the methylene group was around δ 22.41; 22.58; 30.73; 30.97; 51.36; and 75.79 ppm.

## Discussion

In this study, only 1 out of 16 isolate Actinobacteria (InaCC A758) had the potential to produce antimycobacterial compounds, and showed high similarity (99.64%) with *S. parvus* NBRC 14599. As previous reported research, *S. parvus* was known to produce antibacterial compounds, for example *S. parvus* strain C05 could produce granaticin (C_22_H_20_O_10_, MW 440.4), an aromatic polyketide (28). But, there was no report on finding antimycobacterial compound from *S. parvus* strain NBRC 14599. However, *Streptomyces *sp. Av25_2 which was closely related to *S. parvus* NBRC 14599 produced actinomycin D and X2. The actinomycin D was reported to inhibit the growth of *B. subtilis* (29).

The InaCC A758 was indicated to produce actinomycin D from the results of chemical profiling of ethyl acetate extract of the isolate. This may correlate with the presence of the NRPS gene which was 93% related to the NRPS gene of *S. parvulus*. The NRPS gene of *S. parvulus* was known to have a role in actinomycin synthesis. The *S. parvulus* strain 2297, a strain discovered by Waksman in 1940, was known to be able to synthesize actinomycin D, borrelidin, manumacin A, B, and C, and oleficin (30).Other* S. parvulus* strains that also produce actinomycin D, were *S. parvulus* strain RSPSN 2 (31)and *S. parvulus* GQ451836 (32).

The phylogenetic tree of PKS gene of the InaCC A758 showed that it was closely related to the PKS type I borrelidin gene from *S. rochei* and *S. parvulus*. However, the results of the HR-MS analysis did not identify any borrelidin compounds. Borrelidin type I-PKS is known to be identified as a gene group of *S. parvulus* strain Tu4055 which is responsible for the synthesis of the angiogenesis inhibitor borrelidin (C_28_H_43_NO_6_, MW 489.6). In this research, borrelidin could not be expressed from InaCC A758 because of different media used for the culture. *Streptomyces* *rochei* is known to perform borrelidin synthesis using ISP-2 media that has malt and dextrose as a carbon source (33). Meanwhile, *S. parvulus *strain Tu4055 using media that has dextrin and glucose as a carbon source to produce borrelidin (34). It was different from InaCC A758 which uses starch as a carbon source for culture media.

From GC-MS result, the ethyl acetate extract of InaCC A758 produced several peaks that were identified as compounds that have potential as antimicrobials, including ethyl iso-allocholate, which appeared at the peak with a retention time of 21.21; 21.52; 23.47; 23.80 min (Table 2) (35). The ethyl iso-allocholate compound has also been identified from the results of the GC-MS analysis of the extract produced by *S. parvulus* (14). However, ethyl iso-allocholate was not the main secondary metabolite identified in the GC-MS analysis of ethyl acetate extract of InaCC A758.

Other secondary metabolites identified from InaCC A758 ethyl acetate extract with GC-MS analysis were included into the Fatty Acid Methyl Ester (FAME) group, including tetradecanoic acid, 2-hydroxy; 9-octadecanoic acid (Z) -hexyl ester; and 9-hexadecanoic acid. Fatty acid metyl ester has been widely reported to have antimicrobial activity (36), one of which is the 9-hexadecanoic acid compound which also has antimycobacterial activity and other antimicrobial activity (32). Other compounds identified from *S. parvus*, which also identified in InaCC A758 secondary metabolite profile, are hexadecanoic acid, 9,12- octadecadienoic acid (Z, Z) -, methyl ester which are known to have anticancer, anti-carcinogenic, antiatherogenic, anti-oxidants, and anti-inflammatory properties (27).

The long-chain fatty acid structure of alkanoic acid was reported to have an activity to kill *M. smegmatis* and *Mycobacterium bovis*. However, this activity was influenced by the location of the triple bond at the specific carbon chain length of the long-chain fatty acid. The 2-hexadecanoic acid and 2-octadecanoic acid compounds are the most optimal structures to inhibit biosynthesis, extension, and degradation of fatty acids from the mycolic acid structure possessed by *M. smegmatis* and *M. bovis*. Damage to the structure of mycolic acid then results in the death of mycobacterial cells (37).

Meanwhile, the HR-MS analysis of ethyl acetate extract of InaCC A758 denoted that there is a possibility of synthesis of new compounds, due to the low similarity of the chromatogram peaks with compounds in the database. Other similar compounds, instead of dimethenamid, are cyclo (phenylalanyl-prolyl), N1- (2-pyridylmetyl) -4-metylbenzene-1-sulfonamide, dabrafenib, 3- (tert-butyl) -N- (4- (2,3-dihydroimidazole) [2 , 1-b] [1,3] thiazole, and actinomycin D with a match range from 71.6 to 92% database.

The chemical profiling using HR-MS detected several compounds that have not been reported to have antimycobacterial activity, including N-acetyltyramine, orbencarb, and cyclo (leucylprolyl). The N-acetyltyramine, a tyramine alkaloid, is synthesized by *Aspergillus fumigatus*, and has a cytotoxic effect (38). In addition, N-acetyltyramine can be synthesized by *Vibrio alginolyticus* with quorum sensing inhibitor activity (39). Orbencarb was detected with a peak area of ​​1.68% and m/z 257.06183 have a pesticide property (40). Another compound known to have pesticide activity was dimethenamid (41). Then, cyclo (leucylprolyl), which was a pyrroloses quiterpenes, can be synthesized by *Streptomyces *sp. KH-614 which was closely related to *Streptomyces lydicus*, and has antileukemic and anti-vancomycin-resistant enterococci (VRE) activity (42).

The HR-MS analysis of ethyl acetate extract of InaCC A758 only predicted one compound that has ever been reported to have antimycobacterial activity, the actinomycin D. Actinomycin is an antibiotic that has been discovered since 1940 by Selman Waksman, who is also the inventor of streptomycin, from *Actinomyces antibioticus*. Actinomycin has the molecular formula of C_41_H_56_N_8_O_11_, C_37_H_50_N_7_O_10_, or C_36_H_49_N_7_O_9_. ½ H_2_O (43).

Actinomycin is antineoplastic and antibiotic that has a mechanism of action to inhibit cell division (44) and inhibit the ribonucleic acid (RNA) polymerase synthesis (45). The actinomycin D specifically inhibits the pantothenate synthetase (PS) in *M. tuberculosis*. The PS is an enzyme encoded by the *panC* gene that catalyzes the biosynthesis of pantothenate (vitamin B5), which is an important precursor essential for the growth of mycobacteria (46). The IC_50_ value of actinomycin D against PS *M. tuberculosis* was 250.72 ± 39.69 µM (47).

From the purification results of InaCC A758 ethyl acetate extract, two potential compounds were obtained. Compound 1 inhibits the growth of *M. tuberculosis* strain H37Rv with MIC value of 100 µg/ml. The activity of compound 1 in inhibiting the growth of *M. tuberculosis* was extremely low when compared to compound 2 which has a MIC value of 0.78 µg/ml. Until recently, finding new antimycobacterial that could enter the clinical stage was very difficult. Therefore, the discovery of potential compounds, although insignificant when compared to standard drugs, should be appreciated. Compound 1 can be used for further research, for example efforts to increase its activity, by being used in concurrent with nanomaterials such as zinc or silver oxide (48). In addition, compound 1 can be combined with non-antibiotic adjunctive therapies such as naphthoquinone which has been shown to increase the activity of isoniazid and rifampicin, or with oleanolic acid which helps increase membrane permeability and inhibit the DNA polymerase of *M. tuberculosis* (49).

Compound 1 was predicted as a compound similar to dimethenamid (C_12_H_18_ClNO_2_S, MW 275.0723) based on the HR-MS results, as one of the compounds identified with a large peak at a retention time of 15.95 min. Dimethenamid is an herbicide, included in the chloroacetamide group. Technically it has a dark brown color (41). However, compound 1 has a clear brownish color, and when oxidized, it can turn to purple. Actinobacteria are known to produce herbicides or bio pesticides (50), but so far there is no report of dimethenamid production from *Streptomyces sp*. Actinobacteria are reported to produce herbicides other than dimethenamid, including 2,4-dihydro-4- (β-D-ribofuranosyl) -1, 2, 4 (3H) -triazol-3-one by *Actinomadura spp* (51) and ivermectin by *Streptomyces avermitilis* (52).

However, the process of structural elucidation of compound 1 had not finished yet because there were some differences in the interpretation of HR-MS results with the FT-IR and NMR data. Compound 1 was concluded to have a different spectrum from dimethenamid in the peak between 3406 to 3004 cm-1. In addition, the ^13^C-NMR result showed that there were 24 carbon peaks which probably indicated that there were 24 carbon atoms in the compound 1 structure. The presence of phenol or methyl aromatic groups, as shown in NMR result did not completely match with the structure of the predictive compound dimethenamid. Therefore, further research is needed to ascertain the structure of compound 1.

Compound 2 was the most active compound produced by InaCC A758, with MIC value 0.78 µg/ml. It was indicated to be more active, compared to the actinomycin V produced by *Streptomyces capoamus*, with MIC value of 3.125 µg/ml (53). 

The FT-IR results exhibited that the spectra of the compound 2 resembled the IR spectra of the actinomycin D produced by *S. parvulus* RSPSN2. The actinomycin produced peaks at 3421, 2924, 2855, 1741, 1653, 1436, 1124, 1024, 952, and 760 cm-1 (31), which represented primary amine groups, hydroxyl, alkenes, primary amides, and carbonyl. The results of FT-IR spectra analysis of other studies on actinomycin D showed that there was a peak indicating the presence of the –NH group (3500 - 3200 cm-1) and the carbonyl functional group (1744 and 1632 cm-1). The presence of bands in the regions 2927.7 and 2857.8 cm-1, respectively, was caused by asymmetry and symmetry stretching of the C-H bonds in the –CH2 group. In addition, C-H bending vibrations were seen at 1460 and 1370 cm-1 (56).

The ^13^C NMR analysis also provides confirmatory data that compound 2 is actinomycin D. The chemical shift of compound 2 has similarities to that actinomycin that has been found from previous studies (Table 8) (54, 55).

In addition to having antimycobacterial activity, actinomycin D is also known to have an activity to inhibit the growth of methicillin-resistant *Staphylococcus aureus* (MRSA), *Escherichia coli, Candida albicans* (55), streptomycin resistant bacteria such as *Pseudomonas mirabilis, Pseudomonas putida,* and *Bacillus cereus* (31). Apart from having antimicrobial activity, actinomycin D is also reported to have cytotoxic activity. These activities include inhibiting the growth of human cell lines from breast cancer cells (MCF-7), melanoma cells (A375), prostate cancer cells (DU145), lung cancer cells (A549) (57), and inhibit glioma cell proliferation (55).

**Table 1:** Antimycobacterial activity of 16 Actinobacteria isolates from *endophytes* of *Curcuma zedoaria *and mangrove ecosystem in Indonesia and their NRPS/PKS genes detection

No	Isolate Code	Isolate Source	Origin Location	Inhibition of *M. tuberculosis* strains H37Rv (MIC µg/mL)*	NRPS gene	PKS gene
1.	A619	Rhizome, *Curcuma zedoaria*	Bojong Gede, Bogor, West Java	-	-	-
2.	A621	Rhizome, *Curcuma zedoaria*	Bojong Gede, Bogor, West Java	-	+	+
3.	A622	Rhizome, *Curcuma zedoaria*	Bojong Gede, Bogor, West Java	-	+	-
4.	A623	Rhizome, *Curcuma zedoaria*	Bojong Gede, Bogor, West Java	-	-	-
5.	A626	Rhizome, *Curcuma zedoaria*	Bojong Gede, Bogor, West Java	-	-	-
6.	A627	Rhizome, *Curcuma zedoaria*	Bojong Gede, Bogor, West Java	-	-	-
7.	A633	Rhizome, *Curcuma zedoaria*	Bojong Gede, Bogor, West Java	-	+	-
8.	A641	Stem, *Curcuma zedoaria*	Bojong Gede, Bogor, West Java	-	+	-
9.	A753	Sedimen	Pramuka Island, Kepulauan Seribu, DKI Jakarta	-	+	-
10.	A758	Rhizosphere soil (mud)	Pramuka Island, Kepulauan Seribu, DKI Jakarta	+ (25)	+	+
11.	A759	Rhizosphere soil (mud)	Pramuka Island, Kepulauan Seribu, DKI Jakarta	-	+	+
12.	A760	Rhizosphere soil (mud)	Pramuka Island, Kepulauan Seribu, DKI Jakarta	-	-	-
13.	A761	Rhizosphere soil (mud)	Pramuka Island, Kepulauan Seribu, DKI Jakarta	-	+	-
14.	A765	Sand beach	Pramuka Island, Kepulauan Seribu, DKI Jakarta	-	+	+
15.	A766	Sand beach	Pramuka Island, Kepulauan Seribu, DKI Jakarta	-	-	-
16.	A767	Sand beach	Pramuka Island, Kepulauan Seribu, DKI Jakarta	-	-	-

NRPS; non-ribosomal peptide synthetase; PKS: polyketide synthase

**Table 2:** GC-MS compound analysis of crude ethyl acetate extract of InaCC A758

Peak Number	Retention Time (min)	Compound Name	Chemical Formula	Molecular Weight	Similarity Index	Area (%)
1	1.46	1-Nonylcycloheptane	C_16_H_32_	224.432	752	10.47
2	1.51	Nitric Oxide	NO	30.01	626	NC*
3, 4	1.55; 1.57	Methyl Alcohol	CH_4_O	32.04	853	NC*
5	2.01	Methane, oxybis[dichloro-	C_2_H_2_Cl_4_O	183.849	706	4.08
6	2.46	10-Undecenoic acid, octyl ester	C_19_H_36_O_2_	296.50	708	1.18
7	3.04	Tetradecanoic acid, 2-hydroxy-	C_14_H_28_O_3_	244.37	703	2.87
8	4.16	9-Hexadecenoic acid	C_16_H_30_O_2_	254.414	706	0.99
9	4.27	9-octadecenoic acid (Z), hexyl ester	C_24_H_46_O_2_	366.60	671	0.76
10, 11, 13	11.21; 11.27; 12.58	Octadecane, 6-methyl-	C_19_H_40_	268.529	812	39.00
12, 14	12.26; 13.41	Hexadecane, 1,1-bis(dodecyloxy)-	C_40_H_82_O_2_	595.094	792	6.76
15	13.89	1-Hexadecanol, 2-methyl-	C_17_H_36_O	256.474	772	5.44
16	14.92	1-Hexanamine, 2-ethyl-N-(2-ethylhexyl)-	C_16_H_35_N	241.463	694	5.66
17 -24	16.32; 16.73; 17.78; 18.77; 19.39; 19.73; 20.08; 20.64	12-Methyl-E,E-2,13-octadecadien-1-ol	C_19_H_36_O	280.50	794	11.40
25 - 28	21.21; 21.52; 23.47; 23.80	Ethyl-iso-allocholate	C_26_H_44_O_5_	436.60	765	9.86
29	26.49	trans-13-Octadecenoic acid	C_18_H_34_O_2_	282.50	741	1.54

The calculated area was the area of each compound divided by the total area of the chromatogram. *NC (Not Calculated), methyl alcohol as solvent (27)

**Table 3:** Secondary metabolite prediction in InaCC A758 ethyl acetate extract, based on peak chromatogram profile of HR-MS analysis*

Compound Prediction	Chemical Formula	Molecular Weight	Retention Time (Min)	Similarity Index*	% Peak Area
Similar to: Dimethenamid	C_12_H_18_ClNO_2_S	275.0723	15.94	57.7	50.72
Similar to: Cyclo(phenylalanyl-prolyl)	C_6_H_19_N_2_P_3_S	244.0494	15.93	92.0	6.48
Similar to: N1-(2-Pyridylmethyl)-4-methylbenzene-1-sulfonamide	C_13_H_14_N_2_O_2_S	262.0773	15.04	71.6	5.57
N-Acetyltyramine	C_10_H_13_NO_2_	179.0945	3.42	94.1	3.47
Similar to: Dabrafenib	C_13_H_13_N_3_O_3_S	291.0672	13.56	72.7	2.39
Similar to: 4-[2-(2-Nitrobenzoyl)hydrazino]-4-oxobut-2-enoic acid	C_11_H_9_N_3_O_6_	301.0344	15.70	62.2	1.82
Orbencarb	C_12_H_16_ClNOS	257.0618	23.34	79.0	1.68
Similar to: 3-(tert-Butyl)-N-[4-(2,3-dihydroimidazo[2,1-b][1,3]thiazol-6-yl)phenyl]-1-methyl-1H-pyrazole-5-carboxamide	C_12_H_9_N_3_OS	243.0463	22.96	92.0	1.50
Cyclo (leucylprolyl)	C_11_H_18_N_2_O_2_	210.1366	14.27	92.6	1.14
4-hydroxyephedrine	C_10_H_15_NO_2_	163.0995	17.03	74.7	1.11
Similar to: 6'-Methoxy-2'-methyl-3',4',6,8-tetrahydro-2'H-spiro[indeno[4,5-d][1,3]dioxole-7,1'-isoquinoline]-7',8-diol 2'-oxide	C_8_H_9_N_10_P	276.0756	3.76	82.4	0.53
Similar to: Actinomycin D	C_62_H_101_N_2_O_18_P_3_	1254.6285	25.46	79.8	0.42

*Secondary metabolites shown in this table were compounds with high peak area only (> 0.4%)

**Table 4:** ^13^C-NMR data of compound 1

δ (ppm)	δ (ppm)	δ (ppm)
18.18	18.43	57.04
57.33	105.08	106.23
122.48	123.60	123.67
125.92	126.34	138.87
139.21	142.75	148.93
150.16	150.79	154.223
155.25	156.68	158.47
169.75	169.09	206.09

**Table 5:** ^1^H-NMR data of compound 1

δ (ppm)	Splitting	J (Hz)
1.90	Singlet	0.52
2.42	Duplet	14.18
4.15	Duplet	13.97
4.88	Duplet	-
7.46	Multiplet	4.60
8.01	Multiplet	2.88
8.12	Multiplet	1.21
8.39	Singlet	1.00
8.54	Duplet	3.28
8.71	Multiplet	4.56
8.94	Singlet	3.01

**Table 6:** ^1^H-NMR data of compound 2

δ (ppm)	Splitting	J (Hz)
0.78	Duplet	20.44
0.89	Duplet	19.61
0.98	Duplet	19.80
1.14	Duplet	18.07
1.28	Duplet	17.18
2.02	Multiplet	12.32
2.14	Multiplet	31.26
2.59	Multiplet	15.15
2.87	Singlet	16.97
2.96	Singlet	21.05
3.68	Multiplet	12.77
3.99	Multiplet	6.58
4.86	Duplet	0.43
5.24	Double duplet	6.18
6.10	Duplet	5.84
7.45	Duplet	3.36
7.54	Duplet	1.74
8.16	Duplet	2.25

**Table 7:** ^13^C-NMR data of compound 2

δ (ppm)	δ (ppm)	δ (ppm)
7.80	15.17	17.64
18.18	19.51	19.65
19.77	19.99	20.02
21.83	21.89	21.96
23.90	28.50	32.22
32.47	32.99	33.23
35.51	35.65	35.71
39.51	49.15	52.67
56.21	58.52	58.64
72.12	72.28	76.15
76.28	76.41	103.10
103.34	114.48	126.55
129.34	130.83	131.42
133.82	141.98	146.67
147.35	149.11	168.31
168.54	168.74	168.93
170.05	170.11	175.68
175.77	180.62	180.74

**Table 8:** ^13^C NMR data of compound 2 (CD_4_O solvent) compared to actinomycin D (CDCl_3_ solvent)

No	Chemical Shift (ppm) of compound 2	Actinomycin D (54)	Actinomycin D (55)	The Number of Carbons Associated with the Functional Group
1	130.82; 133.82; 126.55; 131.42; 129.34; 141.98; 146.67; 114.48; 180.74; 149.11; 103.34; 147.35	129.20; 132.66; 125.53; 130.03; 127.90; 140.37; 144.92; 113.27; 179.08; 147.29; 102.04; 145.65	129.1; 132.4; 125.7; 130.4; 127.8; 140.5; 145.1; 113.5; 179.1; 147.8; 101.6; 145.9	Signal between ^δ^C 180 and 100 (12 atom C) from phenoxazinone ring
2	168.31; 168.54; 168.74; 168.93; 170.05; 170.11; 175.68; 175.77	166.69; 166.69; 170.46; 170.46; 173.07; 173.07; 173.07; 173.67; 166.69; 166.70; 166.69; 166.62	166.60; 166.59; 168.60; 169.10; 173.30; 173.70; 173.40; 173.50; 166.40, 166.70; 167.70; 167.80	Signal between ^δ^C 173 and 166 (12 atom C) are amide dan carbonyl lacton peptide system
3	15.17; 7.81; 52.67; 56.22; 56.52; 58.64; 32.22; 32.47; 19.51; 19.65; 19.77; 19.93; 23.91; 28.50; 32.96; 33.27; 35.51; 35.65; 35.71; 39.51; 72.12; 72.28; 19.99; 20.02; 21.83; 21.89; 17.64; 18.18	14.01; 7.70; 56.43; 56.59; 58.90; 58.85; 31.52; 31.78; 19.04; 19.14; 19.22; 19.31; 47.37; 47.39; 23.00; 24.67; 31.52; 31.78; 56.43; 56.59; 33.86; 35.00; 51.35; 51.35; 38.98; 39.10; 71.13; 71.12; 27.04; 27.11; 19.04; 19.22; 22.56; 22.64; 17.15; 17.64	15.10; 7.70; 55.20; 54.90; 58.90; 58.70; 31.50; 31.80; 19.00; 19,30; 47.40; 47,70; 23.00; 22.80; 31.00; 31,30; 56.40; 56,60; 35.00; 35.00; 51.30; 51,40; 39.30; 39.20; 71.30; 71.20; 27.00; 27.00; 19.10; 19.10; 21.60; 21.70; 17.30; 17.80	Signal between ^δ^C 72 and 17 (36 atom C) are carbon atom of α from amino acid
4	76.15; 76.28	75.02; 75.13	75.0; 75.10	Signal between ^δ^C 76 and 75 (2 atom C) are oxygenated metilene carbon

## Conclusion

InaCC A758 produced secondary metabolites that inhibit *M. tuberculosis* strain H37Rv. Fractionation and purification of ethyl acetate extract of InaCC A758 identified two antimycobacterial active compounds, namely compound 1 (C_12_H_18_ClNO_2_S; MW 275.0723) which was predicted as a compound similar to dimethenamid with MIC value of 100 µg/ml, and compound 2 (C_62_H_86_N_12_O_16_; m/z 1254.6285), with MIC value of 0.78 µg/ml, which was identified as actinomycin D.
